# Supplementary material for: Determinants of School dropouts among adolescents: Evidence from a longitudinal study in India
Source: PLoS One. 2023 Mar 2;18(3):e0282468. doi: 10.1371/journal.pone.0282468 (PMC9980764; doi:10.1371/journal.pone.0282468)
Supplement: S1 Appendix — (DOCX) [file pone.0282468.s001.docx]

Appendix Table 1. The characteristics at wave 1 of adolescents who were re-interviewed and who were not

| **Baseline Variable** | **Respondents lost to follow up** | **Respondents interviewed in the follow-up sample** | **Mean difference** |  |
| --- | --- | --- | --- | --- |
| Years of education (mean) | 7.33 | 7.37 | 0.04 |  |
| Completed 8 or more years of education (%) | 58.70 | 58.60 | 0.10 |  |
| Currently in School (%) | 57.00 | 64.80 | 7.8*** |  |
| Mothers level of education (mean) | 2.91 | 2.51 | 0.40*** |  |
| Place of residence (%) | 45.20 | 57.50 | 12.3*** |  |
| Social group (% SC\ST) | 21.60 | 24.30 | 2.7*** |  |
| Religion (% Hindu) | 73.70 | 80.00 | 6.3*** |  |
| HH wealth Score (mean) | 22.57 | 21.51 | 1.06*** |  |
| **Total number of respondents** | **4302** | **16292** |  |  |

*** p<0.01, ** p<0.05, * p<0.1
